# Evaluation of a novel, multicomponent anxiety management programme for people with intellectual disability: protocol for a mixed-methods, quasi-experimental feasibility study

**DOI:** 10.1136/bmjopen-2023-078411

**Published:** 2023-09-20

**Authors:** Daniel Acton, Jonathan Williams, Ceri Woodrow, Grace Talbot, Steven Jones, Steven Lane, Sujeet Jaydeokar

**Affiliations:** 1 Learning Disabilty Services, Cheshire and Wirral Partnership NHS Foundation Trust, Chester, UK; 2 Medical School, University of Chester, Chester, UK; 3 Centre for Medical Statistics and Health Evaluation, University of Liverpool, Liverpool, UK

**Keywords:** mental health, feasibility studies, protocols & guidelines, patient-centered care, anxiety disorders

## Abstract

**Introduction:**

Studies have shown some benefits to single approaches to psychological therapies for the treatment of anxiety in people with intellectual disability such as modified cognitive–behavioural therapy and mindfulness. To our knowledge, no studies have used a multicomponent approach for the individual treatment of anxiety-related disorders in this population group. A co-production group of clinical experts and people with intellectual disability has created a novel multicomponent anxiety management programme (MCAMP-ID). The aims of this study are to investigate (1) the feasibility of this approach in reducing anxiety for people with a mild/moderate intellectual disability, (2) the feasibility of outcome measures and (3) the feasibility of completing a future randomised controlled trial of this programme. The data from this feasibility study will be used to inform trial design and to complete power calculation.

**Methods and analysis:**

Sixty people with intellectual disability will be invited to participate in the study across four intellectual disability services within one mental health trust in Northwest England. The specialist services will deliver either treatment as usual (TAU) or the novel intervention (MCAMP-ID). MCAMP-ID comprises of 10 individual sessions delivered by a member of the clinical team once a week for between 10 and 12 weeks. TAU will be based on standard treatment currently delivered to meet the person’s specific needs. The outcomes of the study will be feasibility of recruitment, attrition, adherence to the programme and suitability of outcome measures. A mixed-methods approach will be used to assess outcomes.

**Ethics and dissemination:**

The study received approval from the Research Ethics Committee and Health Research Authority (23/EM/0044) through the Integrated Research Application System (IRAS ID: 315557) in March 2023. Participants will provide informed consent before taking part. Study findings will be presented at conferences and published within a peer-reviewed journal.

**Trial registration number:**

ISRCTN16062949.

**STRENGTHS AND LIMITATIONS OF THIS STUDY**

- Feasibility study to determine if a future definitive trial can be completed.
- A strength is the use of co-production to develop a manualised multicomponent anxiety management programme.
- A further strength is the use of a mixed-methods approach, with qualitative and quantitative analyses.
- A limitation is the anxiety programme manuals have not previously been fully tested in clinical practice, which is the aim of this feasibility trial.

## Introduction

There is a greater incidence of mental health difficulties in people with intellectual disability than in the general population.^1 2^ Anxiety-related issues are the most common^3 4^ Some evidence suggests anxiety difficulties may increase over a person’s life course due to exposure to negative life events which can impact on quality of life.^5 6^ High levels of anxiety in people with intellectual disability is sometimes demonstrated as behaviour of challenge, for example, self-injury, aggression or property damage, since individuals may be unable to effectively communicate or cope with high levels of stress and anxiety.^7^ Subsequently, there can be a tendency for interventions to focus more towards behavioural management strategies.^8^

Anxiety can have a significant effect on quality of life and limit opportunities to engage in social and recreational activities. This can result in people experiencing poor self-esteem, low mood, increased isolation, loneliness and cognitive distortions.^3^ Equally, people with intellectual disability can have difficulty making sense of their own thoughts, with overcoming avoidance behaviours or developing effective self-management strategies.^9^ Psychoeducation is an important component in treatment for anxiety-related difficulties for people with intellectual disability because it helps the individual to understand that they can influence their illness experience and live a better quality of life.^10^ Therefore, the efficacy of psychological therapies is of paramount importance given the prevalence of mental health difficulties in this population group.^11^

In the UK, clinical guidance for the treatment of mental health difficulties in people with intellectual disability highlighted the limited body of evidence for effective psychological therapies^12^ (National Institute for Health and Care Excellence (NICE)). However, there are a small but growing number of studies which have examined the effectiveness of adapted psychological therapies for the treatment of mental health problems.^13^

Studies have examined modified cognitive–behavioural therapy (CBT) for a range of emotional problems for people with intellectual disability.^14^ A feasibility randomised controlled trial (RCT) of CBT for the treatment of depression and anxiety in people with a mild intellectual disability demonstrated limited long-term effectiveness.^15^ A meta-analysis examining CBT for the treatment of a range of mental health problems found limited efficacy for the treatment of anxiety in people with intellectual disability.^13^ More significant and sustained improvements in anxiety and depression symptoms were reported for 12 people with intellectual disability in a study examining a modified transdiagnostic programme of CBT.^16^ Discrepancies may be partly explained by a systematic review finding small sample sizes and lack of methodological rigour in study designs a significant factor in identifying clinical effectiveness within study outcomes.^17^

Studies of psychological therapies such as mindfulness have been found to be effective when people with intellectual disability are provided with adequate support and guidance to practise requisite skills.^13 18 19^ NICE clinical guidelines suggest psychological therapy be adapted for people with intellectual disabilities, identifying CBT, relaxation and graded exposure techniques as recommended treatment for anxiety.^12^ However, despite recommendation, there is no current evidence of any adapted multicomponent therapies for the treatment of anxiety in people with intellectual disability.

Multicomponent therapies combine key therapeutic skills that work together to improve the effectiveness of treatment.^20^ More recently, some success of multicomponent therapies for anxiety in the general adult population has been identified which demonstrates promise for this psychological approach.^21^ There are very few adapted therapies for mental health difficulties that are suitable for people with a moderate intellectual disability as well as those with a milder intellectual impairment.^19 22^

### Co-producing a multicomponent anxiety management programme manual

We have worked with people with intellectual disability with a lived experience of mental health difficulties to co-produce and develop a multicomponent psychological therapy for the treatment of anxiety. A five-phase process (Box 1) was used to guide the adaptation of psychological therapies included within the treatment manuals.^23^

Box 1 **Five-phase process for adapting psychological therapies**

- Phase 1: generating knowledge and collaborating with stakeholders.
- Phase 2: integrating generated information with theory and empirical and clinical knowledge.
- Phase 3: review of culturally adapted clinical intervention by stakeholders and further revision.
- Phase 4: testing the culturally adapted intervention.
- Phase 5: synthesising stakeholder feedback and finalising the culturally adapted intervention.

An initial draft clinician and service user’s manual was subsequently developed with input from specialist clinical staff, service users, their families and carers.^24^ Initial trialling of sessions with people with intellectual disability was completed and feedback allowed modifications to the programme including increasing the number of treatment sessions to 10.

### Aim and objectives

The aim of this study is to discover the feasibility of this approach in reducing anxiety for people with mild or moderate intellectual disability. Additionally, we will establish the feasibility of conducting a future RCT using a multicomponent approach for the treatment of anxiety and development of self-management skills in people with intellectual disability. This will be assessed by examining recruitment, attrition rates, adherence to the treatment programme and National Health Service (NHS) treatment costs.

Our secondary objective is to assess the appropriateness of outcome measures which include the Glasgow Anxiety Scale (GAS-ID),^25^ Hospital Anxiety and Depression Scale (HADS),^26^ adapted WHO Quality of Life (WHOQOL-8)^27^ and Client Satisfaction Questionnaire (CSQ-8)^28^ with the intervention. We will complete power calculation to estimate sample size for future RCT.

## Methods and analysis

### Study design

This feasibility study is a randomised controlled quasi-experimental trial of a novel multicomponent anxiety management intervention in comparison with treatment as usual (TAU). Each arm of the study will have 30 participants and they will be allocated between four sites, with two sites comprising the control group and the other two sites the intervention arm. To reduce selection bias, group allocation will be completed by allocating each team with a number and one investigator placing numbers in an envelope and an independent person randomly selecting numbers for the assignment of groups.

The intervention will be delivered over the duration of 10 weeks to allow for some flexibility to meet the needs of people with severe intellectual disability. Baseline measures will be completed 1 week prior to the intervention, at the end of the intervention (12 weeks) and again at follow-up 8 weeks post-completion to establish any sustained effect on levels of anxiety.

The feasibility trial will be supported by people with intellectual disability who have a lived experience of mental health issues and will form an essential part of the patient and public involvement (PPI) group.

### Study timeline

The study will be completed over 24 months that started on 29 March 2023. Recruitment started on 3 March 2023 and will continue until August 2024. Follow-up assessments, data cleansing and analysis will be completed between September 2024 until March 2025.

### Recruitment and screening

Participants will be recruited from four community intellectual disability services within the Northwest region of England. Participants will be identified from people currently requiring treatment or those referred to specialist intellectual disability services for an anxiety-related difficulty.

Professionals from intellectual disability services will be provided with information on inclusion/exclusion criteria and asked to screen their caseloads to identify potential participants. Participants will be provided with accessible information about the study and given the opportunity to meet a member of the research study team, if they require further information.

Potential participants who are interested in taking part will be asked permission for their name and contact details to be provided to the research study team. A member of the team will contact the person and their family or carer and arrange a visit to assess if they meet the inclusion criteria. If the person meets the eligibility criteria and is willing to participate, then informed consent will be obtained.

Participants will be screened for eligibility using the anxiety component of the Moss-PAS-ID anxiety.^29^ Participants will be eligible for the study if they score 7 or above.

### Sample size

A total of 60 adults with intellectual disabilities will be invited to participate in the study. Sample sizes of between 50 and 60 are considered large enough to allow sufficient power to evaluate feasibility of a definitive RCT.^30^

### Inclusion criteria

- Participants aged over 18 years of age.
- Registered diagnosis of mild or moderate intellectual disability with or without autism.
- Scores above 7 (range 7–18) on the anxiety component of the Moss-PAS-ID. 29
- Required to provide informed consent and sign a declaration form indicating agreement to participate.

### Exclusion criteria

- If they choose not to be involved in the study.
- Have a severe or profound intellectual disability.
- Lack capacity to consent.
- Under the age of 18 years old.
- Participants who score below 7 on the anxiety component of the Moss-PAS-ID.

All decisions regarding participant exclusion will be made by the specialist intellectual disability clinical team.

### Capacity and consent

All participants will be required to have capacity to consent to taking part in the study. This will be assessed, if required, by a member of the research team in line with Mental Capacity Act (2005). Participants will be provided with information about the study in an accessible format and provided with an opportunity to discuss or find out more about the study prior to agreeing to participate.

Participants who agree to participate will be asked to sign a consent form to indicate their agreement to be part of the trial (online supplemental material). If at any time a participant decides they no longer wish to take part in the study, they will be made aware of their right to withdraw by notifying a member of the research team.

10.1136/bmjopen-2023-078411.supp1 Supplementary data

### Intervention group

The novel multicomponent anxiety management programme (MCAMP-ID) intervention will comprise of up to 12 face-to-face sessions using a co-developed novel anxiety management manual for the delivery of treatment sessions. Each session begins and ends with a breathing exercise to support skill learning before the main anxiety management activity session. The first session provides an outline of the programme, selects a location for sessions and establishes ground rules before introducing anxiety and developing a person-centred plan and therapy goals. Table 1 provides an overview of session structure.

**Table 1:** Overview of session structure

Session no	Session plan	Session summary
1	Introduction	Basic introduction to anxiety, setting out the components of the therapy, and practical considerations, goal setting.
2	Healthy mind and body	Simple healthy living principles that provide a foundation to cope with anxiety, for example, diet, exercise and sleep factors.
3	Talking about anxiety	Further psychoeducation about anxiety, for example, anxiety is a normal emotion, physiology and language to describe anxiety.
4	Learning new skills	Practical introduction to mindfulness and self-soothing skills.
5	Working on my anxiety skills	Considering potentially anxiety-producing scenarios (scenario cards) and coping skills that could support.
6	One step at a time	Revisiting personal goals, developing a graded exposure plan and using anxiety skills to facilitate steps.
7	My second step	Review of progress so far, troubleshooting, further practise of anxiety-coping skills, setting next steps towards goals.
8	My third step	Review of progress since the prior session so far, troubleshooting, further practise of anxiety coping skills, setting next steps towards goals.
9	My last step	Review of progress since the prior session and next steps to continue progress after the programme.
10	My anxiety plan	Developing personalised plan to include consolidation of all aspects of the programme including goals, lifestyle factors, anxiety management skills and mindfulness and soothing skills. Provision of separate summary for supporters to help the person continue their progress.

### Control group

TAU for anxiety in people with mild intellectual disability consists of a single component-adapted psychological treatment to develop coping skills. TAU provides support with identifying and linking thoughts with emotions and developing problem-solving skills. These will be provided by community multidisciplinary team members and will be delivered once a week for up to 12 weeks.

### Training of professionals in delivery of anxiety management interventions

Clinicians across all four sites within the NHS trust will receive standardised training on the delivery of the MCAMP-ID programme (intervention group) and a separate training session for single-component therapy (control group). Training will be delivered jointly by people with lived experience (PwLE) and clinical psychologists who are part of the research study team. The training will include using the easy-to-read materials to ensure reasonable adjustments and accessibility of treatment intervention.

### Quantitative measures

Clinical outcome measures will be completed in three stages: baseline, at 12 weeks and 20 weeks (8 weeks post-completion) of the programme (either face to face, telephone or via video link).

### Primary outcome measure

#### Glasgow Anxiety Scale

The GAS-ID is a 27-item self-rating scale for people with intellectual disabilities which is designed to identify cognitive, behavioural and somatic symptoms, which co-present with anxiety disorders.^25^ The GAS-ID is chosen because it provides a robust and validated assessment of a person’s level of anxiety which has been specifically designed for people with intellectual disability.

### Secondary outcome measures

#### Hospital Anxiety and Depression Scale

The HADS is a 14-item interview questionnaire that measures depression, anxiety and severity of the emotional disorder.^26^ The HADS is an effective validated measure that has been chosen to identify any correlation between levels of anxiety and depression in people with intellectual disability.

#### Client Satisfaction Questionnaire

The CSQ-8 is an eight-item measure of client satisfaction eliciting the client’s perception of the mental health services and intervention rated on a 4-point Likert scale.^28^ To understand patients, families and carers on the development, experience of going through the intervention, and acceptability of the intervention and the manual, the CSQ-8 will be completed at end of intervention (12 weeks).

#### WHO Quality of Life Measure

The WHOQOL-8 is an eight-item quality of life measure covering four domains of the original 26-item version.^27^ To measure quality of life, we will use an adapted version of the WHOQOL. This consists of eight questions rated on a 4-point Likert scale.

#### Client Service Receipt Inventory

The Client Service Receipt Inventory (CSRI) is a questionnaire used to estimate service utilisation and will be used to evaluate the cost and use of health resources and services by service users with psychiatric problems.^31^ A modified version of the CSRI will be tailored to the specific context in collaboration with the PPI group and used to capture health service usage, baseline clinical characteristics of participants and adherence to the study.

### Qualitative approach

On completion of the intervention, participants will be invited to attend a focus group or individual interview facilitated by PwLE and a member of the research team. Semistructured interview questions will be used to understand participants’ thoughts and feelings of the anxiety management intervention. All interviews will be audio-recorded and transcribed verbatim. Thematic analysis process described by Braun and Clarke^32^ will be used to analyse the transcripts.

### Quantitative analysis

Data will be analysed using the computer software program SPSS (V.28). The descriptive analysis will focus principally on recruitment rates, characteristics of participants and the function of the programme manuals. The primary outcomes (mean scores and CIs) will be reported at baseline, 12 weeks and 20 weeks (8 weeks post-completion) for both comparative arms. The variability of the primary outcome measure will be used to inform the power calculation and sample size for the definitive RCT. Additionally, we will complete analyses of treatment preference and number of sessions received.

A descriptive analysis of total NHS costs and individual resource use components will be completed.

### Criteria for progression to full RCT

We will consider progressing to a full RCT, if we are able to recruit and retain participants. Therefore, feasibility of recruitment will be a key aspect of this feasibility study. A Stop/Go process will be used to ascertain the percentage of participants recruited to the trial. Go: 100% recruited and at least 80% of eligible participants retained. Review: 50–79% of eligible participants recruited; Stop: <50% participants recruited. With the retention rate of 80%, a sample size of 60 will allow these outcomes to be estimated with an accuracy of ±11%.

### Reporting

**Figure 1:**
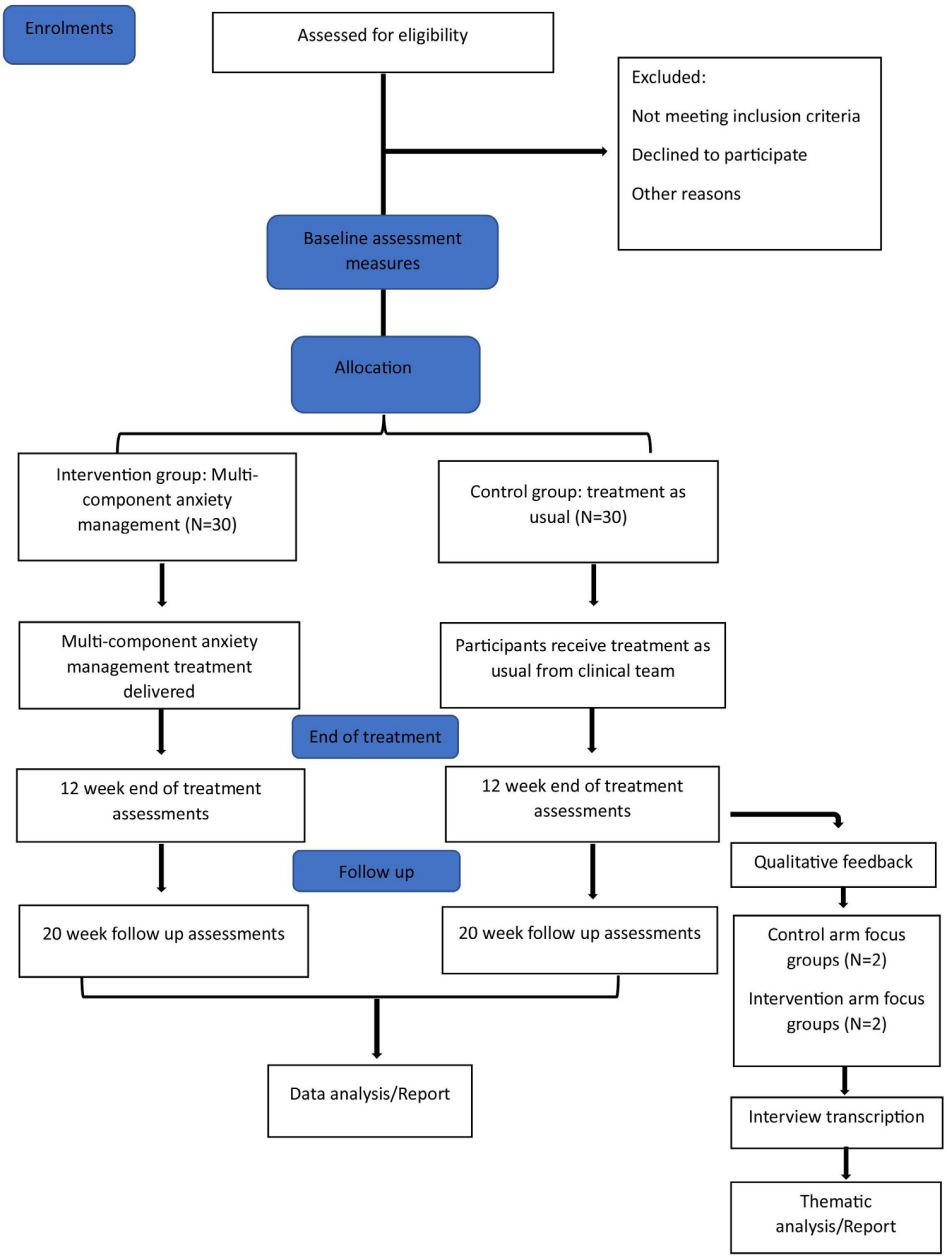
Template for the study flow diagram.

The Consolidated Standards of Reporting Trials (CONSORT) extension for pilot and feasibility studies will be used to guide reporting of the study results and monitor the flow of participants recruited to the study.^33^ Figure 1 is the template for the CONSORT study flow diagram, giving an overview of the study design and planned process of participant recruitment.

### Patient and public involvement

People with intellectual disability have been involved in co-producing and developing the multicomponent anxiety management programme manuals. They will be part of the PwLE group and will continue to play an active role in the feasibility trial by co-developing and designing resources and information leaflets, developing and delivering staff training, co-facilitating focus groups and analysing qualitative data. Additionally, the PwLE group are independent from the sponsor and will chair the trial committee. A participation and engagement practitioner will provide support to the PwLE group throughout the trial, to ensure people are fully supported to engage in all aspects of the study.

## Ethics and dissemination

The study received approval from the Research Ethics Committee and Health Research Authority (23/EM/0044) through the Integrated Research Application System (IRAS ID: 315557) in March 2023. Any amendments to the protocol or procedures will require approval from the Health Research Authority before any changes are made. Participants will be required to provide informed consent before taking part (online supplemental material).

The results of the study will be published in peer-reviewed journals with the findings disseminated at conferences, professional networks, social media and family carer forums. A short film will be made with the PPI group detailing the value of co-production with people who have a lived experience of anxiety in developing psychological interventions.

## Supplementary Material

Reviewer comments

Author's manuscript
