# Supplementary material for: Evaluation of a novel, multicomponent anxiety management programme for people with intellectual disability: protocol for a mixed-methods, quasi-experimental feasibility study
Source: BMJ Open. 2023 Sep 20;13(9):e078411. doi: 10.1136/bmjopen-2023-078411 (PMC10514669; doi:10.1136/bmjopen-2023-078411)
Supplement: Supplementary data [file bmjopen-2023-078411supp001.pdf]

Cheshire and Wirral Partnership
NHS Foundation Trust

IRAS Project ID: 315557

Consent Form

Evaluation of a Novel Multi-component Anxiety Management Programme for the Treatment of Anxiety Disorder in People with Intellectual Disability

Please tick one box		No	Yes
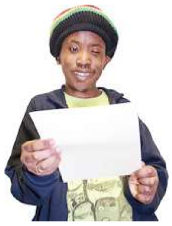	I have read the information sheet about the research<br><br>(version 1.5 date 06/03/2023)		
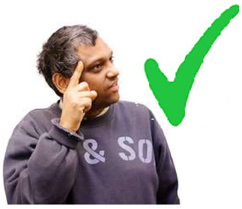	I can understand the information in the information sheet		
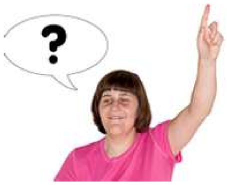	I was able to ask questions if I wanted to		

V1.4 06.03.2023

Please tick one box		No	Yes
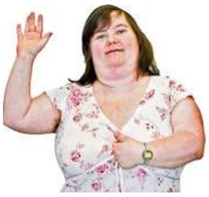	I understand that it is my choice to take part in this study		
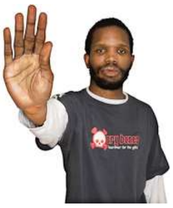	I understand that I can say **No** at any time if I want to stop		
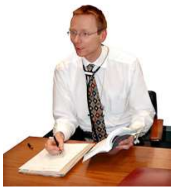	I agree to my GP (doctor) being told I am taking part		
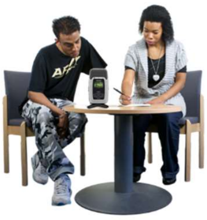	I agree to take part in an interview at the end of the study. I will not repeat what is discussed in the interview.<br><br>(optional)		
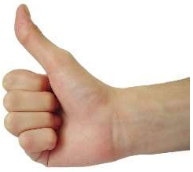	I am happy to take part in the study		

V1.4 06.03.2023

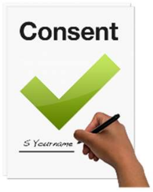

I am happy to take part in the study

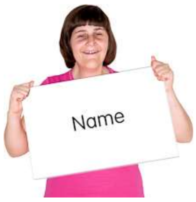

My name

My Signature

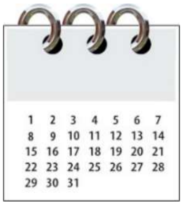

Date

Researchers name

Signature

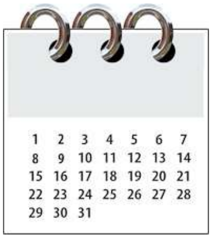

Date

V1.4 06.03.2023
