# Supplementary material for: Evaluation of a novel, multicomponent anxiety management programme for people with intellectual disability: protocol for a mixed-methods, quasi-experimental feasibility study
Source: BMJ Open. 2023 Sep 20;13(9):e078411. doi: 10.1136/bmjopen-2023-078411 (PMC10514669; doi:10.1136/bmjopen-2023-078411)
Supplement: Author's manuscript [file bmjopen-2023-078411.draft_revisions.pdf]

BMJ Open is committed to open peer review. As part of this commitment we make the peer review history of every article we publish publicly available.

When an article is published we post the peer reviewers' comments and the authors' responses online. We also post the versions of the paper that were used during peer review. These are the versions that the peer review comments apply to.

The versions of the paper that follow are the versions that were submitted during the peer review process. They are not the versions of record or the final published versions. They should not be cited or distributed as the published version of this manuscript.

BMJ Open is an open access journal and the full, final, typeset and author-corrected version of record of the manuscript is available on our site with no access controls, subscription charges or pay-per-view fees (http://bmjopen.bmj.com).

If you have any questions on BMJ Open's open peer review process please email info.bmjopen@bmj.com

BMJ Open

Evaluation of a novel multi-component anxiety management programme: a mixed methods quasi-experimental feasibility study

Journal:	*BMJ Open*
Manuscript ID	bmjopen-2023-078411
Article Type:	Protocol
Date Submitted by the Author:	01-Aug-2023
Complete List of Authors:	Acton, Danny; Cheshire and Wirral Partnership NHS Foundation Trust, Learning disabilty services<br>Williams, Jonathan; Cheshire and Wirral Partnership NHS Foundation Trust, Intellectual disability services<br>Woodrow, Ceri; Cheshire and Wirral Partnership NHS Foundation Trust, Intellectual disability services<br>Talbot, Grace; Cheshire and Wirral Partnership NHS Foundation Trust, Intellectual disability services<br>Jones, Steven; University of Chester,<br>Lane, Steven; University of Liverpool, Centre for medical statistics and health eval<br>Jaydeokar , Sujeet; Cheshire and Wirral Partnership NHS Foundation Trust; University of Chester, Medical School
Keywords:	MENTAL HEALTH, Feasibility Studies, Protocols & guidelines < HEALTH SERVICES ADMINISTRATION & MANAGEMENT, Patient-Centered Care, Anxiety disorders < PSYCHIATRY

SCHOLARONE™
Manuscripts

Evaluation of a novel multi-component anxiety management programme: a mixed methods quasi-experimental feasibility study

Abstract

Introduction: Studies have shown some benefits to single approaches to psychological therapies for the treatment of anxiety in people with intellectual disability such as modified cognitive behaviour therapy and mindfulness. To our knowledge, no studies have used a multi-component approach for the individual treatment of anxiety related disorders in this population group. A co-production group of clinical experts and people with intellectual disability have created a novel multi-component anxiety management programme (M CAMP-ID). Therefore, the aims of this study are: i) to discover the feasibility of this approach in reducing anxiety for people with a mild/moderate intellectual disability, ii) the feasibility of outcome measures, and iii) feasibility of completing a future randomised control trial of this programme. The data from this feasibility study will be used to inform trial design and to complete power calculation.

Methods and analysis: Sixty people with intellectual disability will be invited to participate in the study across four intellectual disability services within one mental health trust in Northwest England. The specialist services will be randomly selected to deliver either treatment as usual (TAU) or the novel intervention (M CAMP-ID). M CAMP-ID comprises of ten individual sessions delivered by a member of the clinical team once a week for between 10 – 12 weeks. TAU will be based on standard treatment currently delivered to meet the person's specific needs. The outcomes of the study will be feasibility of recruitment, attrition, adherence to the programme and suitability of outcome measures. A mixed method approach will be used to assess outcomes.

Strengths and limitations of this study

- Feasibility study to determine if future definitive trial can be completed
- A strength is the use of co-production to develop a manualised multi-component anxiety management programme.
- A further strength is the use of mixed methods approach using both qualitative and quantitative analysis.

- A limitation is the anxiety programme manuals have not been fully tested in clinical practice which is the aim of this feasibility trial.

Introduction

There is a greater incidence of mental health difficulties in people with intellectual disability than in the general population (1, 2). Anxiety related issues are the most common (3, 4) Some evidence suggests anxiety difficulties may increase over a person's life course due to exposure to negative life events which can impact on quality of life (5, 6). High levels of anxiety in people with intellectual disability is sometimes demonstrated as behaviour of challenge, for example self-injury, aggression, or property damage since individuals may be unable to effectively communicate or cope with high levels of stress and anxiety (7). Subsequently, there can be a tendency for interventions to focus more towards behavioural management strategies (8).

Anxiety can have a significant effect on quality of life and limit opportunities to engage in social and recreational activities. This can result in people experiencing poor self-esteem, low mood, increased isolation, loneliness and cognitive distortions (3). Equally, people with intellectual disability can have difficulty making sense of their own thoughts, with overcoming avoidance behaviours, or developing effective self-management strategies (9). Psychoeducation is an important component in treatment for anxiety related difficulties for people with intellectual disability because it helps the individual to understand that they can influence their illness experience and live a better quality of life (10). Therefore, the efficacy of psychological therapies is of paramount importance given the prevalence of mental health difficulties in this population group (11).

In the United Kingdom (UK), clinical guidance for the treatment of mental health problems in people with intellectual disability highlighted the limited body of evidence for effective psychological therapies (12) (NICE). However, there are a small but growing number of studies which have examined the effectiveness of adapted psychological therapies for the treatment of mental health problems (13).

For peer review only

Studies have examined modified Cognitive Behaviour Therapy (CBT) for a range of emotional problems for people with intellectual disability (14). A feasibility randomised control trial of CBT for the treatment of depression and anxiety in people with a mild intellectual disability demonstrated limited long-term effectiveness (15). A meta-analysis examining CBT for the treatment of a range of mental health problems found limited efficacy for the treatment of anxiety in people with intellectual disability (13). More significant and sustained improvements in anxiety and depression symptoms were reported for twelve people with intellectual disability in a study examining a modified trans-diagnostic programme of CBT (16). Discrepancies may be partly explained by a systematic review finding small sample sizes and lack of methodological rigor in study designs a significant factor in identifying clinical effectiveness within study outcomes (17).

There is some evidence to suggest individual rather than group therapy is more effective in this population group (18). Moreover, studies of psychological therapies such as mindfulness have been found to be effective when people with intellectual disability are provided with adequate support and guidance to practice requisite skills (13, 19). NICE clinical guidelines suggest psychological therapy be adapted for people with intellectual disabilities identifying CBT, relaxation and graded exposure techniques as recommended treatment for anxiety (12). However, despite recommendation there is no current evidence of any adapted multi-component therapies for the treatment of anxiety in people with intellectual disability.

Multi-component therapies combine key therapeutic skills that work together to improve the effectiveness of treatment (20). More recently, some success of multi-component therapies for anxiety in the general adult population has been identified which demonstrates promise for this psychological approach (21). There are also very few adapted therapies for mental health difficulties that are suitable for people with a moderate intellectual disability as well as those with a milder intellectual impairment (18, 22).

Co-producing a multi-component anxiety management programme manuals

We have worked with people with intellectual disability with a lived experience of mental health difficulties to co-produce and develop a multi-component psychological therapy for

For peer review only

the treatment of anxiety. A five-stage process (box 1) was used to guide the adaptation of psychological therapies included within the treatment manuals (23).

Box 1 shows a five-stage process for adapting psychological therapies

Insert box 1

An initial draft clinician and service user's manual was subsequently developed with input from a range of specialist clinical staff, service users, their families and carers (24). Initial trialling of sessions with people with intellectual disability was completed and feedback allowed modifications to the programme including increasing the number treatment sessions to ten.

Aim and Objectives

The aim of this study is to discover the feasibility of this approach in reducing anxiety for people with mild or moderate intellectual disability. Additionally we will establish the feasibility of conducting a future randomised control trial using a multi component approach for the treatment of anxiety and development of self-management skills in people with intellectual disability. This will be assessed by examining recruitment, attrition rates, adherence to the treatment programme and NHS treatment costs.

Our secondary objective is to assess the appropriateness of outcome measures which include the Glasgow Anxiety Scale (25), Hospital Anxiety and Depression Scale (26), adapted World Health Organisation Quality of Life (27), and Client Satisfaction Questionnaire (28) with the intervention. We will also complete power calculation to estimate sample size for future randomised control trial.

Research Plan / Methods

Design

This is a feasibility study for a single blind randomised controlled quasi-experimental trial of a novel multi-component anxiety management intervention in comparison to treatment as

For peer review only

usual (TAU). Each arm of the study will have 30 participants and they will be allocated between four sites with two sites comprising the control group and the other two sites the intervention arm. To reduce selection bias, group allocation will be completed by allocating each team with a number and one investigator placing numbers in an envelope and an independent person randomly selecting numbers for the assignment of groups.

The intervention will be delivered over a duration of 10 weeks to allow for some flexibility to meet the needs of people with severe intellectual disability. Baseline measures will be completed 1 week prior to the intervention, at the end of the intervention (12 weeks) and again at follow-up 8 weeks post completion to establish any sustained effect on levels of anxiety.

The feasibility trial will be supported by people with intellectual disability who have a lived experience of mental health issues and will form an essential part of the Patient and Public Involvement group.

Recruitment and screening

Participants will be recruited from four community intellectual disability services within the Northwest region of England. Participants will be identified from people currently requiring treatment or those referred to specialist intellectual disability services for an anxiety related difficulty.

Professionals from intellectual disability services will be provided with information on inclusion/exclusion criteria and asked to screen their caseloads to identify potential participants. Participants will be provided with accessible information about the study and given the opportunity to meet a member of the research study team, if they require further information.

Potential participants who are interested in taking part will be asked permission for their name and contact details to be provided to the research study team. A member of the team will contact the person and their family or carer and arrange a visit to assess if they meet the inclusion criteria. If the person meets the eligibility criteria and is willing to participate, then informed consent will be obtained.

For peer review only

Participants will be screened for eligibility using the anxiety component of the MOSS-Pas-ID anxiety (29). Participants will be eligible for the study if they score seven or above.

Sample size

A total of 60 adults with intellectual disabilities will be invited to participate in the study. Sample sizes of between 50-60 are considered large enough to allow sufficient power to evaluate feasibility of a definitive RCT (30).

Inclusion criteria

- Participants aged over 18 years of age.
- Registered diagnosis of mild or moderate intellectual disability with or without autism.
- Scores above seven (range 7-18) on the anxiety component on the MOSS Pas-ID (Moss et al., 1998).
- Required to provide informed consent and sign a declaration form indicating agreement to participate.

7.2 Exclusion criteria

- If they choose not to be involved in the study.
- Have a severe or profound intellectual disability.
- Lack capacity to consent.
- Under the age of 18 years old.
- Participants who score below 7 on the anxiety component of the Moss PAS-ID.

All decisions regarding participant exclusion will be made by the specialist intellectual disability clinical team.

Capacity and consent

All participants will be required to have capacity to consent to taking part in the study. This will be assessed, if required, by a member of the research team in line with Mental Capacity

For peer review only

Act (2005). Participants will be provided with information about the study in an accessible format and provided with an opportunity to discuss or find out more about the study prior to agreeing to participate.

Participants who agree to participate will be asked to sign a consent form to indicate their agreement to be part of trial.

Intervention group

The novel Multi-Component Anxiety Management Programme (M CAMP-ID) intervention will comprise of up to 12 face-to-face sessions using a co-developed novel anxiety management manual for the delivery of treatment sessions. Each session begins and ends with a breathing exercise to support skill learning before the main anxiety management activity session. The first session provides an outline of the programme, selects a location for sessions, and establishes ground rules before introducing anxiety and developing a person-centred plan and therapy goals. Table 1 provides an overview of session structure.

Insert table 1

Control Group

Treatment as usual (TAU) for anxiety in people with mild intellectual disability consists of a single component adapted psychological treatment to develop coping skills. TAU provides support with identifying and linking thoughts with emotions and developing problem-solving skills. These will be provided by community multidisciplinary team members and will be delivered once a week for up to 12 weeks.

Training of professionals in delivery of anxiety management interventions

Clinicians across all four sites within the NHS Trust will receive standardised training on the delivery of the MCAMP-ID programme (intervention group) and a separate training session for single component therapy (control group). Training will be delivered jointly by people with lived experience (PwLE) and clinical psychologists who are part of the research study

For peer review only

team. The training will include using the easy read materials to ensure reasonable adjustments and accessibility of treatment intervention.

Ethical approval

The study has received approval from the Research Ethics Committee (REC) and Health Research Authority (23/EM/0044) in March 2023. Any amendments to the protocol or procedures will require approval from the HRA before any changes are made.

Quantitative measures

Clinical outcome measures will be completed in three stages, baseline, at 12 weeks and 20 weeks (8 weeks post-completion) of the programme (either face to face, telephone, or via video-link).

Primary outcome measure

Glasgow Anxiety scale (GAS-ID) is a 27 item self-rating scale for people with intellectual disabilities which is designed to identify cognitive, behavioural, and somatic symptoms, which co-present with anxiety disorders (25). The GAS-ID is chosen because it provides a robust and validated assessment of a person's level of anxiety which has been specifically designed for people with intellectual disability.

Secondary outcome measures

Hospital Anxiety and Depression Scale (HADS) is a 14-item interview questionnaire that measures depression, anxiety, and the severity of the emotional disorder (26). The HADS is an effective validated measure has been chosen to identify any correlation between levels of anxiety and depression in people with intellectual disability.

Client Satisfaction Questionnaire (CSQ-8): is an eight item measure of client satisfaction eliciting the client's perception of the mental health services and intervention which is rated on a four point Likert scale (28). To understand patients, families and carers on the development, experience of going through the intervention, and acceptability of the intervention and the manual CSQ-8 will be completed at end of intervention 12 weeks.

For peer review only

World Health Organisation Quality of Life Measure (WHOQOL-8) is a short eight item quality of life measure covering four domains of the original 26 item version (27)**.** To measure quality of life we will use an adapted version of the WHOQOL measure. This consists of eight questions which is rated using a four-point Likert scale.

Client Service Receipt Inventory (CSRI) is a questionnaire used to estimate service utilisation and will be used to evaluate the cost and use of health resources and services by service users with psychiatric problems (31). A modified version of the CSRI will be tailored to the specific context in collaboration with the PPI group and used yo capture health service usage and baseline clinical characteristics of participants.

Qualitative approach

On completion of the intervention participants will be invited to attend a focus group or individual interview facilitated by PwLE and a member of the research team. Semi-structured interview questions will be used to understand participant's thoughts and feeling of the anxiety management intervention. All interviews will be audio recorded and transcribed verbatim. Thematic analysis process described by (32) will be used to analyse the transcripts.

Patient and public involvement (PPI)

People with intellectual disability have been involved in co-producing and developing the multi-component anxiety management programme manuals. They will be part of the PwLE group and will continue to play an active role in the feasibility trial by co-developing and designing resources, information leaflets, developing and delivering staff training, co-facilitating focus groups and analysing qualitative data. Additionally, the PwLE group will chair the trial committee. A Participation and Engagement Practitioner will provide support to the PwLE group throughout the trial, to ensure people are fully supported to engage in all aspects of the study.

Quantitative Analysis

For peer review only

Data will be analysed using computer software programme SPSS (v28). The descriptive analysis will focus principally on recruitment rates, characteristics of participants, and the function of the programme manuals. The primary outcomes (mean scores and confidence intervals) will be reported at baseline, 12 weeks and 20 weeks (8 weeks post-completion) for both comparative arms. The variability of the primary outcome measure will be used to inform the power calculation and sample size for the definitive RCT. Additionally, we will complete analyses of treatment preference and number of sessions received.

A descriptive analysis of total NHS costs and individual resource use components will be completed.

Criteria for progression to full randomised control trial

We will consider progressing to a full randomised control trial if we are able to recruit and retain participants. Therefore, feasibility of recruitment will be a key aspect of this feasibility study. A Stop/Go process will be used to ascertain the percentage of participants recruited to the trial. Go: 100% recruited and at least 80% of eligible participants retained. Review: 50-79% of eligible participants recruited, Stop: <50% participants recruited. With the retention rate of 80%, a sample size of 60 will allow these outcomes to be estimated with an accuracy of +/-11%.

A Consolidated Standards of Reporting Trials (CONSORT) will be used to monitor the flow of participants recruited to the study and provides an overview of the trial design (33) (see figure 1 for study flow diagram).

Dissemination

The results of the study will be published in peer reviewed journals with the findings disseminated at conferences, professional networks, social media and family carer forums. A short film will be made with the PPI group detailing the value of co-production with people who have a lived experience of anxiety in developing psychological interventions.

For peer review only

References

1. Cooper S-A, Smiley E, Morrison J, Williamson A, Allan L. Mental ill-health in adults with intellectual disabilities: prevalence and associated factors. British Journal of Psychiatry. 2007;190(1):27-35.
2. Shimoyama M, Iwasa K, Sonoyama S. The prevalence of mental health problems in adults with intellectual disabilities in Japan, associated factors and mental health service use: The prevalence of mental health problems in Japan. Journal of intellectual disability research. 2018;62(11):931-40.
3. Reid KA, Smiley E, Cooper SA. Prevalence and associations of anxiety disorders in adults with intellectual disabilities. Journal of intellectual disability research. 2011;55(2):172-81.
4. Smiley E. Epidemiology of mental health problems in adults with learning disability: an update. Advances in psychiatric treatment : the Royal College of Psychiatrists' journal of continuing professional development. 2005;11(3):214-22.
5. Tsakanikos E, Bouras N, Costello H, Holt G. Multiple exposure to life events and clinical psychopathology in adults with intellectual disability. Social Psychiatry and Psychiatric Epidemiology. 2007;42(1):24-8.
6. Wigham S, Taylor JL, Hatton C. A prospective study of the relationship between adverse life events and trauma in adults with mild to moderate intellectual disabilities: Life events and trauma: a prospective study. Journal of intellectual disability research. 2014;58(12):1131-40.
7. Pruijssers AC, van Meijel B, Maaskant M, Nijssen W, van Achterberg T. The relationship between challenging behaviour and anxiety in adults with intellectual disabilities: a literature review: Challenging behaviour and anxiety. Journal of intellectual disability research. 2014;58(2):162-71.
8. Westlake F, Hassiotis A, Unwin G, Totsika V. The role of behaviour problems in screening for mental ill-health in adults with intellectual disability. The European journal of psychiatry. 2021;35(2):122-5.
9. Unwin G, Tsimopoulou I, Kroese BS, Azmi S. Effectiveness of cognitive behavioural therapy (CBT) programmes for anxiety or depression in adults with intellectual disabilities: A review of the literature. Research in Developmental Disabilities. 2016;51:60-75.
10. Jagla M, Augustin M, Baumeister A, Franke G. Psychoeducation for people with intellectual disabilities and anxiety or schizophrenic disorders. European Health Psychologist. 2016:793-.
11. Bhaumik S, Gangadharan S, Hiremath A, Russell PSS. Psychological treatments in intellectual disability: the challenges of building a good evidence base. The British Journal of Psychiatry. 2011;198(6):428-30.
12. National Institute for Health and Care Excellence. Mental health problems in people with learning disabilities: prevention, assessment and management. NICE guideline [NG54]. 2016.
13. Koslowski N, Klein K, Arnold K, Kösters M, Schützwohl M, Salize HJ, et al. Effectiveness of interventions for adults with mild to moderate intellectual disabilities and mental health problems: systematic review and meta-analysis. British journal of psychiatry. 2016;209(6):469-74.
14. Graser J, Göken J, Lyons N, Ostermann T, Michalak J. Cognitive-behavioral therapy for adults with intellectual disabilities: A meta-analysis. Clinical psychology (New York, NY). 2022;29(3):227-42.
15. Hassiotis A, Serfaty M, Azam K, Strydom A, Blizard R, Romeo R, et al. Manualised Individual Cognitive Behavioural Therapy for mood disorders in people with mild to moderate intellectual disability: A feasibility randomised controlled trial. Journal of affective disorders. 2013;151(1):186-95.
16. Lindsay WR, Tinsley S, Beail N, Hastings RP, Jahoda A, Taylor JL, et al. A preliminary controlled trial of a trans-diagnostic programme for cognitive behaviour therapy with adults with intellectual disability. Journal of intellectual disability research. 2015;59(4):360-9.
17. Dagnan D, Jackson I, Eastlake L. A systematic review of cognitive behavioural therapy for anxiety in adults with intellectual disabilities. Journal of intellectual disability research. 2018;62(11):974-91.

18. Vereenooghe L, Langdon PE. Psychological therapies for people with intellectual disabilities: A systematic review and meta-analysis. Research in developmental disabilities. 2013;34(11):4085-102.
19. Idusohan-Moizer H, Sawicka A, Dendle J, Albany M. Mindfulness-based cognitive therapy for adults with intellectual disabilities: an evaluation of the effectiveness of mindfulness in reducing symptoms of depression and anxiety. Journal of intellectual disability research. 2015;59(2):93-104.
20. Antoine P, Dauvier B, Andreotti E, Congard A. Individual differences in the effects of a positive psychology intervention: Applied psychology. Personality and individual differences. 2018;122:140-7.
21. Heniks T, Schotanus-dijkstra M, Hassankhan A, de Jong J, Bohlmeijer E. The Efficacy of Multi-component Positive Psychology Interventions: A Systematic Review and Meta-analysis of Randomized Controlled Trials. Journal of happiness studies. 2020;21(1):357-90.
22. Tapp K, Vereenooghe L, Hewitt O, Scripps E, Gray KM, Langdon PE. Psychological therapies for people with intellectual disabilities: An updated systematic review and meta-analysis. Comprehensive psychiatry. 2023;122:152372-.
23. Hwang W-C. The Formative Method for Adapting Psychotherapy (FMAP): A Community-Based Developmental Approach to Culturally Adapting Therapy. Professional psychology, research and practice. 2009;40(4):369-77.
24. Acton DJ, Waites R, Jaydeokar S, Jones S. Co-design and development of a multi-component anxiety management programme for people with an intellectual disability. Advances in mental health and intellectual disabilities. 2023;17(1):26-36.
25. Mindham J, Espie CA. Glasgow Anxiety Scale for people with an Intellectual Disability (GAS-ID): development and psychometric properties of a new measure for use with people with mild intellectual disability. Journal of Intellectual Disability Research. 2003;47(1):22-30.
26. Zigmond AS, Snaith RP. The hospital anxiety and depression scale. Acta Psychiatr Scand. 1983;67(6):361-70.
27. Skevington SM, Lotfy M, O'Connell KA. The World Health Organization's WHOQOL-BREF quality of life assessment: psychometric properties and results of the international field trial. A report from the WHOQOL group. Quality of life Research. 2004;13:299-310.
28. Attkisson CC, Zwick R. The client satisfaction questionnaire. Psychometric properties and correlations with service utilization and psychotherapy outcome. Evaluation and program planning. 1982;5(3):233.
29. Moss S, Prosser H, Costello H, Simpson N, Patel P, Rowe S, et al. Reliability and validity of the PAS-ADD Checklist for detecting psychiatric disorders in adults with intellectual disability. Journal of Intellectual Disability Research. 1998;42(2):173-83.
30. Sim J, Lewis M. The size of a pilot study for a clinical trial should be calculated in relation to considerations of precision and efficiency. Journal of clinical epidemiology. 2012;65(3):301-8.
31. Beecham J, Knapp M. Costing psychiatric interventions. Measuring mental health needs1992. p. 163-83.
32. Braun V, Clarke V. Using thematic analysis in psychology. Qualitative research in psychology. 2006;3(2):77-101.
33. Eldridge SM, Chan CL, Campbell MJ, Bond CM, Hopewell S, Thabane L, et al. CONSORT 2010 statement: extension to randomised pilot and feasibility trials. BMJ (Online). 2016;355:i5239-i.

Authors' Contributions

DA and SJ are joint chief investigators and responsible for the management of the study. JW, CW, SL and SJ contributed to the study design and provide statistical input and are co-

For peer review only

applicants. GT contributed to the implementation of the study in clinical practice. All the authors were involved in writing, revising the manuscript and agreeing on the final version. DJ and SJ will have access to the final trial dataset.

Funding

This project is funded by the National Institute of Health and Care Research (NIHR) under its Research for Patient Benefit (RfPB) Programme (Grant Reference Number: NIHR 204370). The views expressed are those of the author(s) and not necessarily those of the NIHR or the Department of Health and Social Care.

For peer review only

Box 1

Phase 1: Generating knowledge and collaborating with stakeholders
Phase 2: Integrating generated information with theory and empirical and clinical Knowledge
Phase 3: Review of culturally adapted clinical intervention by stakeholders and further Revision
Phase 4: Testing the culturally adapted intervention
Phase 5: Synthesizing stakeholder feedback and finalizing the culturally adapted intervention

For peer review only

Figure 1. Study flow diagram

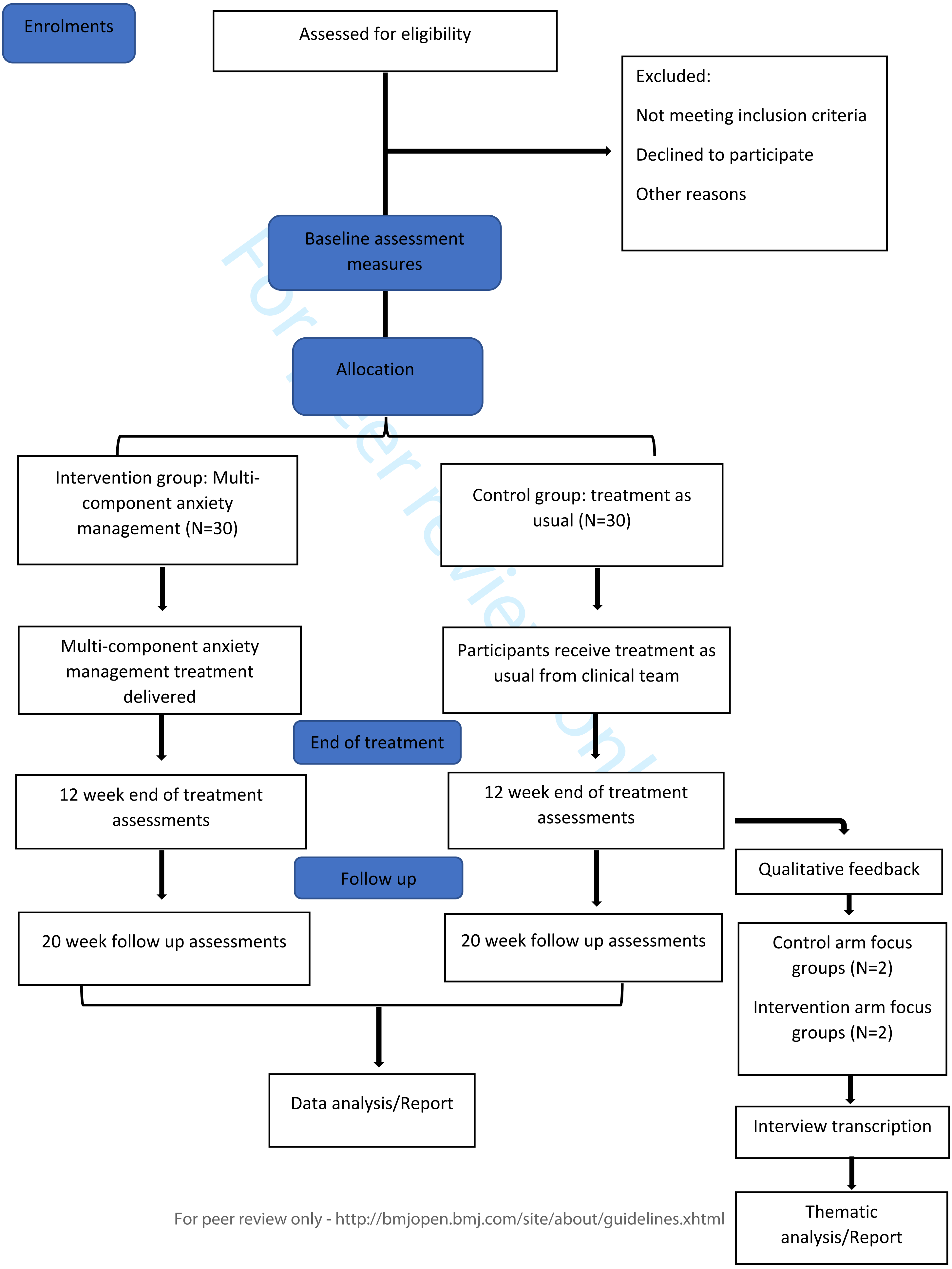

Table 1 overview of session structure:

Session no	Session plan	Session Summary
**1**	Introduction	Basic introduction to anxiety, setting out the components of the therapy, and practical considerations, goal setting.
**2**	Healthy mind and body	Simple healthy living principles that provide a foundation to cope with anxiety, e.g., diet, exercise, and sleep factors.
**3**	Talking about anxiety	Further psychoeducation about anxiety, e.g., anxiety is a normal emotion, physiology, and language to describe anxiety.
**4**	Learning new skills	Practical introduction to mindfulness and self-soothing skills.
**5**	Working on my anxiety skills	Considering potentially anxiety-producing scenarios (scenario cards) and coping skills that could support.
**6**	One step at a time	Revisiting personal goals, developing a graded exposure plan and using anxiety skills to facilitate steps.
**7**	My second step	Review of progress so far, troubleshooting, further practise of anxiety coping skills, setting next steps towards goals.
**8**	My third step	Review of progress since the prior session far, troubleshooting, further practise of anxiety coping skills, setting next steps towards goals
**9**	My last step	Review of progress since the prior session and next steps to continue progress after the programme.
**10**	My anxiety plan	Developing personalised plan to include: consolidation of all aspects of the programme including goals, lifestyle factors, anxiety management skills and mindfulness and soothing skills. Provision of separate summary for supporters to help the person continue their progress.

BMJ Open

Evaluation of a novel, multi-component, anxiety management programme for people with intellectual disability: protocol for a mixed-methods, quasi-experimental feasibility study

Journal:	*BMJ Open*
Manuscript ID	bmjopen-2023-078411.R1
Article Type:	Protocol
Date Submitted by the Author:	08-Aug-2023
Complete List of Authors:	Acton, Danny; Cheshire and Wirral Partnership NHS Foundation Trust, Learning disabilty services<br>Williams, Jonathan; Cheshire and Wirral Partnership NHS Foundation Trust, Intellectual disability services<br>Woodrow, Ceri; Cheshire and Wirral Partnership NHS Foundation Trust, Intellectual disability services<br>Talbot, Grace; Cheshire and Wirral Partnership NHS Foundation Trust, Intellectual disability services<br>Jones, Steven; University of Chester,<br>Lane, Steven; University of Liverpool, Centre for medical statistics and health eval<br>Jaydeokar , Sujeet; Cheshire and Wirral Partnership NHS Foundation Trust; University of Chester, Medical School
**Primary Subject Heading**:	Mental health
Secondary Subject Heading:	Health services research
Keywords:	MENTAL HEALTH, Feasibility Studies, Protocols & guidelines < HEALTH SERVICES ADMINISTRATION & MANAGEMENT, Patient-Centered Care, Anxiety disorders < PSYCHIATRY

SCHOLARONE™
Manuscripts

Evaluation of a novel, multi-component, anxiety management programme for people with intellectual disability: protocol for a mixed-methods, quasi-experimental feasibility study

Abstract

Introduction: Studies have shown some benefits to single approaches to psychological therapies for the treatment of anxiety in people with intellectual disability such as modified cognitive behaviour therapy and mindfulness. To our knowledge, no studies have used a multi-component approach for the individual treatment of anxiety related disorders in this population group. A co-production group of clinical experts and people with intellectual disability have created a novel multi-component anxiety management programme (M CAMP-ID). The aims of this study are to investigate (i) the feasibility of this approach in reducing anxiety for people with a mild/moderate intellectual disability, (ii) the feasibility of outcome measures, and (iii) the feasibility of completing a future randomised control trial of this programme. The data from this feasibility study will be used to inform trial design and to complete power calculation.

Methods and analysis: Sixty people with intellectual disability will be invited to participate in the study across four intellectual disability services within one mental health trust in Northwest England. The specialist services will deliver either treatment as usual (TAU) or the novel intervention (M CAMP-ID). M CAMP-ID comprises of ten individual sessions delivered by a member of the clinical team once a week for between 10 – 12 weeks. TAU will be based on standard treatment currently delivered to meet the person's specific needs. The outcomes of the study will be feasibility of recruitment, attrition, adherence to the programme and suitability of outcome measures. A mixed-method approach will be used to assess outcomes.

Ethics and dissemination: The study received approval from the Research Ethics Committee and Health Research Authority (23/EM/0044) through the Integrated Research Application System (IRAS ID: 315557) in March 2023. Participants will provide informed consent before taking part. Study findings will be presented at conferences and published within a peer-reviewed journal.

Trial registration number: ISRCTN16062949.

For peer review only

Strengths and limitations of this study

- Feasibility study to determine if a future definitive trial can be completed.
- A strength is the use of co-production to develop a manualised multi-component anxiety management programme.
- A further strength is the use of a mixed-methods approach, with qualitative and quantitative analysis.
- A limitation is the anxiety programme manuals have not previously been fully tested in clinical practice, which is the aim of this feasibility trial.

INTRODUCTION

There is a greater incidence of mental health difficulties in people with intellectual disability than in the general population (1, 2). Anxiety related issues are the most common (3, 4) Some evidence suggests anxiety difficulties may increase over a person's life course due to exposure to negative life events which can impact on quality of life (5, 6). High levels of anxiety in people with intellectual disability is sometimes demonstrated as behaviour of challenge, for example self-injury, aggression, or property damage since individuals may be unable to effectively communicate or cope with high levels of stress and anxiety (7). Subsequently, there can be a tendency for interventions to focus more towards behavioural management strategies (8).

Anxiety can have a significant effect on quality of life and limit opportunities to engage in social and recreational activities. This can result in people experiencing poor self-esteem, low mood, increased isolation, loneliness, and cognitive distortions (3). Equally, people with intellectual disability can have difficulty making sense of their own thoughts, with overcoming avoidance behaviours, or developing effective self-management strategies (9). Psychoeducation is an important component in treatment for anxiety related difficulties for people with intellectual disability because it helps the individual to understand that they can influence their illness experience and live a better quality of life (10). Therefore, the efficacy

For peer review only

of psychological therapies is of paramount importance given the prevalence of mental health difficulties in this population group (11).

In the United Kingdom (UK), clinical guidance for the treatment of mental health difficulties in people with intellectual disability highlighted the limited body of evidence for effective psychological therapies (12) (NICE). However, there are a small but growing number of studies which have examined the effectiveness of adapted psychological therapies for the treatment of mental health problems (13).

Studies have examined modified Cognitive Behaviour Therapy (CBT) for a range of emotional problems for people with intellectual disability (14). A feasibility randomised control trial of CBT for the treatment of depression and anxiety in people with a mild intellectual disability demonstrated limited long-term effectiveness (15). A meta-analysis examining CBT for the treatment of a range of mental health problems found limited efficacy for the treatment of anxiety in people with intellectual disability (13). More significant and sustained improvements in anxiety and depression symptoms were reported for twelve people with intellectual disability in a study examining a modified trans-diagnostic programme of CBT (16). Discrepancies may be partly explained by a systematic review finding small sample sizes and lack of methodological rigor in study designs a significant factor in identifying clinical effectiveness within study outcomes (17).

Studies of psychological therapies such as mindfulness have been found to be effective when people with intellectual disability are provided with adequate support and guidance to practice requisite skills (13, 18, 19). NICE clinical guidelines suggest psychological therapy be adapted for people with intellectual disabilities, identifying CBT, relaxation and graded exposure techniques as recommended treatment for anxiety (12). However, despite recommendation there is no current evidence of any adapted multi-component therapies for the treatment of anxiety in people with intellectual disability.

Multi-component therapies combine key therapeutic skills that work together to improve the effectiveness of treatment (20). More recently, some success of multi-component therapies for anxiety in the general adult population have been identified which

For peer review only

demonstrates promise for this psychological approach (21). There are very few adapted therapies for mental health difficulties that are suitable for people with a moderate intellectual disability as well as those with a milder intellectual impairment (19, 22).

Co-producing a multi-component anxiety management programme manuals

We have worked with people with intellectual disability with a lived experience of mental health difficulties to co-produce and develop a multi-component psychological therapy for the treatment of anxiety. A five-phase process (Box 1) was used to guide the adaptation of psychological therapies included within the treatment manuals (23).

Box 1. Five-phase process for adapting psychological therapies

Phase 1: Generating knowledge and collaborating with stakeholders<br><br>Phase 2: Integrating generated information with theory and empirical and clinical Knowledge<br><br>Phase 3: Review of culturally adapted clinical intervention by stakeholders and further Revision<br><br>Phase 4: Testing the culturally adapted intervention<br><br>Phase 5: Synthesizing stakeholder feedback and finalizing the culturally adapted Intervention

An initial draft clinician and service user's manual was subsequently developed with input from specialist clinical staff, service users, their families and carers (24). Initial trialling of sessions with people with intellectual disability was completed and feedback allowed modifications to the programme including increasing the number of treatment sessions to ten.

Aim and objectives

The aim of this study is to discover the feasibility of this approach in reducing anxiety for people with mild or moderate intellectual disability. Additionally we will establish the feasibility of conducting a future randomised control trial using a multi component

For peer review only

approach for the treatment of anxiety and development of self-management skills in people with intellectual disability. This will be assessed by examining recruitment, attrition rates, adherence to the treatment programme and NHS treatment costs.

Our secondary objective is to assess the appropriateness of outcome measures which include the Glasgow Anxiety Scale (25), Hospital Anxiety and Depression Scale (26), adapted World Health Organisation Quality of Life (27), and Client Satisfaction Questionnaire (28) with the intervention. We will complete power calculation to estimate sample size for future randomised control trial.

METHODS AND ANALYSIS

Study design

This is a feasibility study for a single-blind, randomised controlled quasi-experimental trial of a novel multi-component anxiety management intervention in comparison to treatment as usual (TAU). Each arm of the study will have 30 participants and they will be allocated between four sites with two sites comprising the control group and the other two sites the intervention arm. To reduce selection bias, group allocation will be completed by allocating each team with a number and one investigator placing numbers in an envelope and an independent person randomly selecting numbers for the assignment of groups.

The intervention will be delivered over a duration of 10 weeks to allow for some flexibility to meet the needs of people with severe intellectual disability. Baseline measures will be completed 1 week prior to the intervention, at the end of the intervention (12 weeks) and again at follow-up 8 weeks post completion to establish any sustained effect on levels of anxiety.

The feasibility trial will be supported by people with intellectual disability who have a lived experience of mental health issues and will form an essential part of the Patient and Public Involvement group.

Study timeline

For peer review only

The study will be completed over 24 months started on 29th March 2023. Recruitment started on 31st March 2023 and will continue until August 2024. Follow up assessments, data cleansing and analysis will be completed between September 2024 until March 2025.

Recruitment and screening

Participants will be recruited from four community intellectual disability services within the Northwest region of England. Participants will be identified from people currently requiring treatment or those referred to specialist intellectual disability services for an anxiety related difficulty.

Professionals from intellectual disability services will be provided with information on inclusion/exclusion criteria and asked to screen their caseloads to identify potential participants. Participants will be provided with accessible information about the study and given the opportunity to meet a member of the research study team, if they require further information.

Potential participants who are interested in taking part will be asked permission for their name and contact details to be provided to the research study team. A member of the team will contact the person and their family or carer and arrange a visit to assess if they meet the inclusion criteria. If the person meets the eligibility criteria and is willing to participate, then informed consent will be obtained.

Participants will be screened for eligibility using the anxiety component of the MOSS-Pas-ID anxiety (29). Participants will be eligible for the study if they score seven or above.

Sample size

A total of 60 adults with intellectual disabilities will be invited to participate in the study. Sample sizes of between 50-60 are considered large enough to allow sufficient power to evaluate feasibility of a definitive RCT (30).

Inclusion criteria

- Participants aged over 18 years of age.
- Registered diagnosis of mild or moderate intellectual disability with or without autism.

For peer review only

- Scores above seven (range 7-18) on the anxiety component on the MOSS Pas-ID (Moss et al., 1998).
- Required to provide informed consent and sign a declaration form indicating agreement to participate.

Exclusion criteria

- If they choose not to be involved in the study.
- Have a severe or profound intellectual disability.
- Lack capacity to consent.
- Under the age of 18 years old.
- Participants who score below 7 on the anxiety component of the Moss PAS-ID.

All decisions regarding participant exclusion will be made by the specialist intellectual disability clinical team.

Capacity and consent

All participants will be required to have capacity to consent to taking part in the study. This will be assessed, if required, by a member of the research team in line with Mental Capacity Act (2005). Participants will be provided with information about the study in an accessible format and provided with an opportunity to discuss or find out more about the study prior to agreeing to participate.

Participants who agree to participate will be asked to sign a consent form to indicate their agreement to be part of the trial (Supplemental Material). If at any time a participant decides they no longer wish to take part in the study, they will be made aware of their right to withdraw by notifying a member of the research team.

Intervention group

The novel Multi-Component Anxiety Management Programme (M CAMP-ID) intervention will comprise of up to 12 face-to-face sessions using a co-developed novel anxiety

For peer review only

management manual for the delivery of treatment sessions. Each session begins and ends

Session no	Session plan	Session Summary
**1**	Introduction	Basic introduction to anxiety, setting out the components of the therapy, and practical considerations, goal setting.
**2**	Healthy mind and body	Simple healthy living principles that provide a foundation to cope with anxiety, e.g., diet, exercise, and sleep factors.
**3**	Talking about anxiety	Further psychoeducation about anxiety, e.g., anxiety is a normal emotion, physiology, and language to describe anxiety.
**4**	Learning new skills	Practical introduction to mindfulness and self-soothing skills.
**5**	Working on my anxiety skills	Considering potentially anxiety-producing scenarios (scenario cards) and coping skills that could support.
**6**	One step at a time	Revisiting personal goals, developing a graded exposure plan and using anxiety skills to facilitate steps.
**7**	My second step	Review of progress so far, troubleshooting, further practise of anxiety coping skills, setting next steps towards goals.
**8**	My third step	Review of progress since the prior session far, troubleshooting, further practise of anxiety coping skills, setting next steps towards goals
**9**	My last step	Review of progress since the prior session and next steps to continue progress after the programme.
**10**	My anxiety	Developing personalised plan to include consolidation of all aspects of

with a breathing exercise to support skill learning before the main anxiety management activity session. The first session provides an outline of the programme, selects a location for sessions, and establishes ground rules before introducing anxiety and developing a person-centred plan and therapy goals. Table 1 provides an overview of session structure.

plan	the programme including goals, lifestyle factors, anxiety management skills and mindfulness and soothing skills. Provision of separate summary for supporters to help the person continue their progress.

Table 1. Overview of session structure

Control group

TAU for anxiety in people with mild intellectual disability consists of a single component adapted psychological treatment to develop coping skills. TAU provides support with identifying and linking thoughts with emotions and developing problem-solving skills. These will be provided by community multidisciplinary team members and will be delivered once a week for up to 12 weeks.

Training of professionals in delivery of anxiety management interventions

Clinicians across all four sites within the NHS Trust will receive standardised training on the delivery of the MCAMP-ID programme (intervention group) and a separate training session for single component therapy (control group). Training will be delivered jointly by people with lived experience (PwLE) and clinical psychologists who are part of the research study team. The training will include using the easy read materials to ensure reasonable adjustments and accessibility of treatment intervention.

Quantitative measures

Clinical outcome measures will be completed in three stages, baseline, at 12 weeks and 20 weeks (8 weeks post-completion) of the programme (either face to face, telephone, or via video-link).

Primary outcome measure

Glasgow Anxiety scale (GAS-ID) is a 27 item self-rating scale for people with intellectual disabilities which is designed to identify cognitive, behavioural, and somatic symptoms, which co-present with anxiety disorders (25). The GAS-ID is chosen because it provides a robust and validated assessment of a person's level of anxiety which has been specifically designed for people with intellectual disability.

For peer review only

Secondary outcome measures

Hospital Anxiety and Depression Scale (HADS) is a 14-item interview questionnaire that measures depression, anxiety, and the severity of the emotional disorder (26). The HADS is an effective validated measure has been chosen to identify any correlation between levels of anxiety and depression in people with intellectual disability.

Client Satisfaction Questionnaire (CSQ-8) is an eight item measure of client satisfaction eliciting the client's perception of the mental health services and intervention which is rated on a four point Likert scale (28). To understand patients, families and carers on the development, experience of going through the intervention, and acceptability of the intervention and the manual CSQ-8 will be completed at end of intervention (12 weeks).

World Health Organisation Quality of Life Measure (WHOQOL-8) is a eight item quality of life measure covering four domains of the original 26 item version (27)**.** To measure quality of life we will use an adapted version of the WHOQOL measure. This consists of eight questions which is rated using a four-point Likert scale.

Client Service Receipt Inventory (CSRI) is a questionnaire used to estimate service utilisation and will be used to evaluate the cost and use of health resources and services by service users with psychiatric problems (31). A modified version of the CSRI will be tailored to the specific context in collaboration with the PPI group and used to capture health service usage, baseline clinical characteristics of participants and adherence to the study.

Qualitative approach

On completion of the intervention participants will be invited to attend a focus group or individual interview facilitated by PwLE and a member of the research team. Semi-structured interview questions will be used to understand participant's thoughts and feelings of the anxiety management intervention. All interviews will be audio recorded and transcribed verbatim. Thematic analysis process described by (32) will be used to analyse the transcripts.

For peer review only

Quantitative analysis

Data will be analysed using computer software programme SPSS (v28). The descriptive analysis will focus principally on recruitment rates, characteristics of participants, and the function of the programme manuals. The primary outcomes (mean scores and confidence intervals) will be reported at baseline, 12 weeks and 20 weeks (8 weeks post-completion) for both comparative arms. The variability of the primary outcome measure will be used to inform the power calculation and sample size for the definitive RCT. Additionally, we will complete analyses of treatment preference and number of sessions received.

A descriptive analysis of total NHS costs and individual resource use components will be completed.

Criteria for progression to full randomised control trial

We will consider progressing to a full randomised control trial if we are able to recruit and retain participants. Therefore, feasibility of recruitment will be a key aspect of this feasibility study. A Stop/Go process will be used to ascertain the percentage of participants recruited to the trial. Go: 100% recruited and at least 80% of eligible participants retained. Review: 50-79% of eligible participants recruited, Stop: <50% participants recruited. With the retention rate of 80%, a sample size of 60 will allow these outcomes to be estimated with an accuracy of +/-11%.

Reporting

The CONSORT extension for pilot and feasibility studies will be used to guide reporting of the study results monitor the flow of participants recruited to the study (33). Figure 1 is the template CONSORT study flow diagram, giving an overview of the study design and planned process of participant recruitment.

Patient and public involvement

For peer review only

People with intellectual disability have been involved in co-producing and developing the multi-component anxiety management programme manuals. They will be part of the PwLE group and will continue to play an active role in the feasibility trial by co-developing and designing resources, information leaflets, developing and delivering staff training, co-facilitating focus groups and analysing qualitative data. Additionally, the PwLE group are independent from the sponsor and will chair the trial committee. A Participation and Engagement Practitioner will provide support to the PwLE group throughout the trial, to ensure people are fully supported to engage in all aspects of the study.

ETHICS AND DISSEMINATION

The study received approval from the Research Ethics Committee and Health Research Authority (23/EM/0044) through the Integrated Research Application System (IRAS ID: 315557) in March 2023. Any amendments to the protocol or procedures will require approval from the HRA before any changes are made. Participants will be required to provide informed consent before taking part (Supplemental Material).

The results of the study will be published in peer-reviewed journals with the findings disseminated at conferences, professional networks, social media, and family carer forums. A short film will be made with the PPI group detailing the value of co-production with people who have a lived experience of anxiety in developing psychological interventions.

*** ***

Contributors

DA and SJ are joint chief investigators and responsible for the management of the study. JW, CW, SL and SLJ contributed to the study design and provide statistical input and are co-applicants. GT contributed to the implementation of the study in clinical practice. All the authors were involved in writing, revising the manuscript, and agreeing on the final version. DJ and SJ will have access to the final trial dataset.

Competing interests

For peer review only

The authors declare no conflicts of interests.

Funding

This project is funded by the National Institute of Health and Care Research (NIHR) under its Research for Patient Benefit (RfPB) Programme (Grant Reference Number: NIHR 204370). The views expressed are those of the author(s) and not necessarily those of the NIHR or the Department of Health and Social Care.

References

1. Cooper S-A, Smiley E, Morrison J, Williamson A, Allan L. Mental ill-health in adults with intellectual disabilities: prevalence and associated factors. British Journal of Psychiatry. 2007;190(1):27-35.
2. Shimoyama M, Iwasa K, Sonoyama S. The prevalence of mental health problems in adults with intellectual disabilities in Japan, associated factors and mental health service use: The prevalence of mental health problems in Japan. Journal of intellectual disability research. 2018;62(11):931-40.
3. Reid KA, Smiley E, Cooper SA. Prevalence and associations of anxiety disorders in adults with intellectual disabilities. Journal of intellectual disability research. 2011;55(2):172-81.
4. Smiley E. Epidemiology of mental health problems in adults with learning disability: an update. Advances in psychiatric treatment : the Royal College of Psychiatrists' journal of continuing professional development. 2005;11(3):214-22.
5. Tsakanikos E, Bouras N, Costello H, Holt G. Multiple exposure to life events and clinical psychopathology in adults with intellectual disability. Social Psychiatry and Psychiatric Epidemiology. 2007;42(1):24-8.
6. Wigham S, Taylor JL, Hatton C. A prospective study of the relationship between adverse life events and trauma in adults with mild to moderate intellectual disabilities: Life events and trauma: a prospective study. Journal of intellectual disability research. 2014;58(12):1131-40.
7. Pruijssers AC, van Meijel B, Maaskant M, Nijssen W, van Achterberg T. The relationship between challenging behaviour and anxiety in adults with intellectual disabilities: a literature review: Challenging behaviour and anxiety. Journal of intellectual disability research. 2014;58(2):162-71.
8. Westlake F, Hassiotis A, Unwin G, Totsika V. The role of behaviour problems in screening for mental ill-health in adults with intellectual disability. The European journal of psychiatry. 2021;35(2):122-5.
9. Unwin G, Tsimopoulou I, Kroese BS, Azmi S. Effectiveness of cognitive behavioural therapy (CBT) programmes for anxiety or depression in adults with intellectual disabilities: A review of the literature. Research in Developmental Disabilities. 2016;51:60-75.
10. Jagla M, Augustin M, Baumeister A, Franke G. Psychoeducation for people with intellectual disabilities and anxiety or schizophrenic disorders. European Health Psychologist. 2016:793-.
11. Bhaumik S, Gangadharan S, Hiremath A, Russell PSS. Psychological treatments in intellectual disability: the challenges of building a good evidence base. The British Journal of Psychiatry. 2011;198(6):428-30.
12. National Institute for Health and Care Excellence. Mental health problems in people with learning disabilities: prevention, assessment and management. NICE guideline [NG54]. 2016.
13. Koslowski N, Klein K, Arnold K, Kösters M, Schützwohl M, Salize HJ, et al. Effectiveness of interventions for adults with mild to moderate intellectual disabilities and mental health problems: systematic review and meta-analysis. British journal of psychiatry. 2016;209(6):469-74.

For peer review only

14. Graser J, Göken J, Lyons N, Ostermann T, Michalak J. Cognitive-behavioral therapy for adults with intellectual disabilities: A meta-analysis. Clinical psychology (New York, NY). 2022;29(3):227-42.
15. Hassiotis A, Serfaty M, Azam K, Strydom A, Blizard R, Romeo R, et al. Manualised Individual Cognitive Behavioural Therapy for mood disorders in people with mild to moderate intellectual disability: A feasibility randomised controlled trial. Journal of affective disorders. 2013;151(1):186-95.
16. Lindsay WR, Tinsley S, Beail N, Hastings RP, Jahoda A, Taylor JL, et al. A preliminary controlled trial of a trans-diagnostic programme for cognitive behaviour therapy with adults with intellectual disability. Journal of intellectual disability research. 2015;59(4):360-9.
17. Dagnan D, Jackson I, Eastlake L. A systematic review of cognitive behavioural therapy for anxiety in adults with intellectual disabilities. Journal of intellectual disability research. 2018;62(11):974-91.
18. Idusohan-Moizer H, Sawicka A, Dendle J, Albany M. Mindfulness-based cognitive therapy for adults with intellectual disabilities: an evaluation of the effectiveness of mindfulness in reducing symptoms of depression and anxiety. Journal of intellectual disability research. 2015;59(2):93-104.
19. Vereenooghe L, Langdon PE. Psychological therapies for people with intellectual disabilities: A systematic review and meta-analysis. Research in developmental disabilities. 2013;34(11):4085-102.
20. Antoine P, Dauvier B, Andreotti E, Congard A. Individual differences in the effects of a positive psychology intervention: Applied psychology. Personality and individual differences. 2018;122:140-7.
21. Heniks T, Schotanus-dijkstra M, Hassankhan A, de Jong J, Bohlmeijer E. The Efficacy of Multi-component Positive Psychology Interventions: A Systematic Review and Meta-analysis of Randomized Controlled Trials. Journal of happiness studies. 2020;21(1):357-90.
22. Tapp K, Vereenooghe L, Hewitt O, Scripps E, Gray KM, Langdon PE. Psychological therapies for people with intellectual disabilities: An updated systematic review and meta-analysis. Comprehensive psychiatry. 2023;122:152372-.
23. Hwang W-C. The Formative Method for Adapting Psychotherapy (FMAP): A Community-Based Developmental Approach to Culturally Adapting Therapy. Professional psychology, research and practice. 2009;40(4):369-77.
24. Acton DJ, Waites R, Jaydeokar S, Jones S. Co-design and development of a multi-component anxiety management programme for people with an intellectual disability. Advances in mental health and intellectual disabilities. 2023;17(1):26-36.
25. Mindham J, Espie CA. Glasgow Anxiety Scale for people with an Intellectual Disability (GAS-ID): development and psychometric properties of a new measure for use with people with mild intellectual disability. Journal of Intellectual Disability Research. 2003;47(1):22-30.
26. Zigmond AS, Snaith RP. The hospital anxiety and depression scale. Acta Psychiatr Scand. 1983;67(6):361-70.
27. Skevington SM, Lotfy M, O'Connell KA. The World Health Organization's WHOQOL-BREF quality of life assessment: psychometric properties and results of the international field trial. A report from the WHOQOL group. Quality of life Research. 2004;13:299-310.
28. Attkisson CC, Zwick R. The client satisfaction questionnaire. Psychometric properties and correlations with service utilization and psychotherapy outcome. Evaluation and program planning. 1982;5(3):233.
29. Moss S, Prosser H, Costello H, Simpson N, Patel P, Rowe S, et al. Reliability and validity of the PAS-ADD Checklist for detecting psychiatric disorders in adults with intellectual disability. Journal of Intellectual Disability Research. 1998;42(2):173-83.
30. Sim J, Lewis M. The size of a pilot study for a clinical trial should be calculated in relation to considerations of precision and efficiency. Journal of clinical epidemiology. 2012;65(3):301-8.
31. Beecham J, Knapp M. Costing psychiatric interventions. Measuring mental health needs1992. p. 163-83.

For peer review only

32. Braun V, Clarke V. Using thematic analysis in psychology. Qualitative research in psychology. 2006;3(2):77-101.
33. Eldridge SM, Chan CL, Campbell MJ, Bond CM, Hopewell S, Thabane L, et al. CONSORT 2010 statement: extension to randomised pilot and feasibility trials. BMJ (Online). 2016;355:i5239-i.

FIGURE TITLE

Figure 1. Template study flow diagram

For peer review only

Figure 1. Study flow diagram

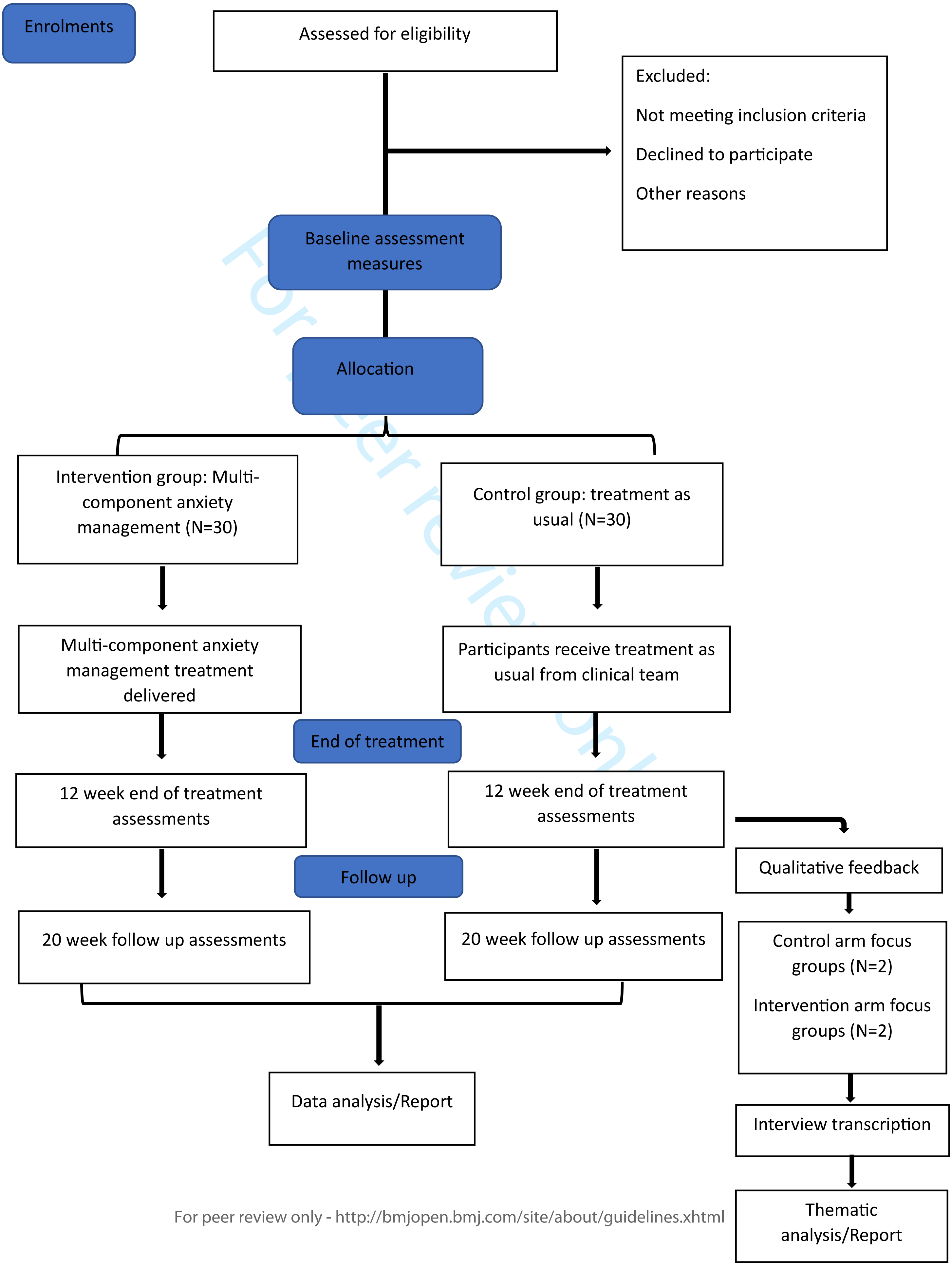

Cheshire and Wirral Partnership
NHS Foundation Trust

IRAS Project ID: 315557

Consent Form

Evaluation of a Novel Multi-component Anxiety Management Programme for the Treatment of Anxiety Disorder in People with Intellectual Disability

Please tick one box		No	Yes
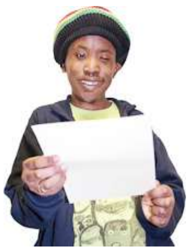	I have read the information sheet about the research<br><br>(version 1.5 date 06/03/2023)		
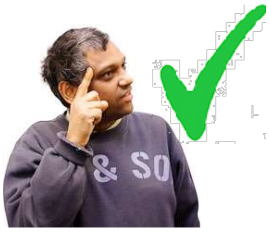	I can understand the information in the information sheet		
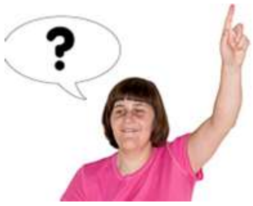	I was able to ask questions if I wanted to		

For peer review only - http://bmjopen.bmj.com/site/about/guidelines.xhtml

Please tick one box		No	Yes
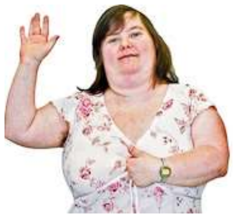	I understand that it is my choice to take part in this study		
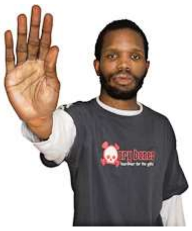	I understand that I can say **No** at any time if I want to stop		
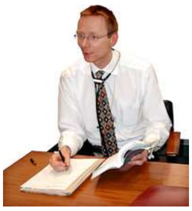	I agree to my GP (doctor) being told I am taking part		
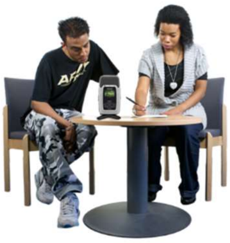	I agree to take part in an interview at the end of the study. I will not repeat what is discussed in the interview. (optional)		
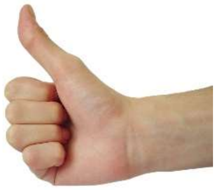	I am happy to take part in the study		

For peer review only

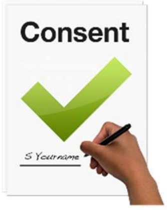

I am happy to take part in the study

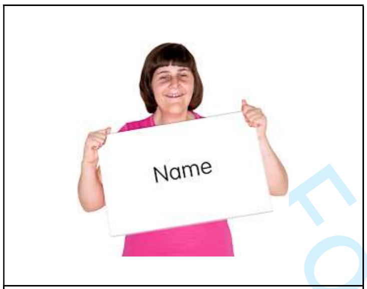

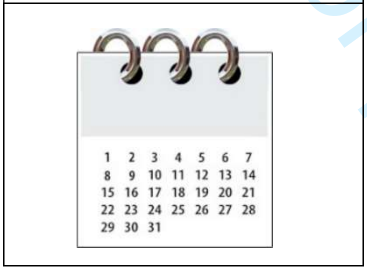

My name

My Signature

Date

Researchers name

Signature

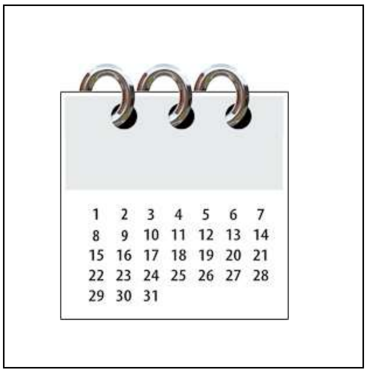

Date

V1.4 06.03.2023

For peer review only - http://bmjopen.bmj.com/site/about/guidelines.xhtml

SPIRIT

STANDARD PROTOCOL ITEMS: RECOMMENDATIONS FOR INTERVENTIONAL TRIALS

SPIRIT 2013 Checklist: Recommended items to address in a clinical trial protocol and related documents*

Section/item	Item No	Description	Line Number
**Administrative information**			
Title	1	Descriptive title identifying the study design, population, interventions, and, if applicable, trial acronym	1
Trial registration	2a	Trial identifier and registry name. If not yet registered, name of intended registry	46
	2b	All items from the World Health Organization Trial Registration Data Set	46
Protocol version	3	Date and version identifier	
Funding	4	Sources and types of financial, material, and other support	345
Roles and responsibilities	5a	Names, affiliations, and roles of protocol contributors	334
	5b	Name and contact information for the trial sponsor	
	5c	Role of study sponsor and funders, if any, in study design; collection, management, analysis, and interpretation of data; writing of the report; and the decision to submit the report for publication, including whether they will have ultimate authority over any of these activities	
	5d	Composition, roles, and responsibilities of the coordinating centre, steering committee, endpoint adjudication committee, data management team, and other individuals or groups overseeing the trial, if applicable (see Item 21a for data monitoring committee)	
**Introduction**			
Background and rationale	6a	Description of research question and justification for undertaking the trial, including summary of relevant studies (published and unpublished) examining benefits and harms for each intervention	48
	6b	Explanation for choice of comparators	
Objectives	7	Specific objectives or hypotheses	120

For peer review only

Trial design	8	Description of trial design including type of trial (eg, parallel group, crossover, factorial, single group), allocation ratio, and framework (eg, superiority, equivalence, noninferiority, exploratory)	135
**Methods: Participants, interventions, and outcomes**			
Study setting	9	Description of study settings (eg, community clinic, academic hospital) and list of countries where data will be collected. Reference to where list of study sites can be obtained	160
Eligibility criteria	10	Inclusion and exclusion criteria for participants. If applicable, eligibility criteria for study centres and individuals who will perform the interventions (eg, surgeons, psychotherapists)	182
Interventions	11a	Interventions for each group with sufficient detail to allow replication, including how and when they will be administered	213
	11b	Criteria for discontinuing or modifying allocated interventions for a given trial participant (eg, drug dose change in response to harms, participant request, or improving/worsening disease)	208
	11c	Strategies to improve adherence to intervention protocols, and any procedures for monitoring adherence (eg, drug tablet return, laboratory tests)	
	11d	Relevant concomitant care and interventions that are permitted or prohibited during the trial	213
Outcomes	12	Primary, secondary, and other outcomes, including the specific measurement variable (eg, systolic blood pressure), analysis metric (eg, change from baseline, final value, time to event), method of aggregation (eg, median, proportion), and time point for each outcome. Explanation of the clinical relevance of chosen efficacy and harm outcomes is strongly recommended	244
Participant timeline	13	Time schedule of enrolment, interventions (including any run-ins and washouts), assessments, and visits for participants. A schematic diagram is highly recommended (see Figure)	308
Sample size	14	Estimated number of participants needed to achieve study objectives and how it was determined, including clinical and statistical assumptions supporting any sample size calculations	178

For peer review only - http://bmjopen.bmj.com/site/about/guidelines.xhtml

Recruitment	15	Strategies for achieving adequate participant enrolment to reach target sample size	298
**Methods: Assignment of interventions (for controlled trials)**			
Allocation:			
Sequence generation	16a	Method of generating the allocation sequence (eg, computer-generated random numbers), and list of any factors for stratification. To reduce predictability of a random sequence, details of any planned restriction (eg, blocking) should be provided in a separate document that is unavailable to those who enrol participants or assign interventions	
Allocation concealment mechanism	16b	Mechanism of implementing the allocation sequence (eg, central telephone; sequentially numbered, opaque, sealed envelopes), describing any steps to conceal the sequence until interventions are assigned	
Implementation	16c	Who will generate the allocation sequence, who will enrol participants, and who will assign participants to interventions	
Blinding (masking)	17a	Who will be blinded after assignment to interventions (eg, trial participants, care providers, outcome assessors, data analysts), and how	135
	17b	If blinded, circumstances under which unblinding is permissible, and procedure for revealing a participant's allocated intervention during the trial	
**Methods: Data collection, management, and analysis**			
Data collection methods	18a	Plans for assessment and collection of outcome, baseline, and other trial data, including any related processes to promote data quality (eg, duplicate measurements, training of assessors) and a description of study instruments (eg, questionnaires, laboratory tests) along with their reliability and validity, if known. Reference to where data collection forms can be found, if not in the protocol	238
	18b	Plans to promote participant retention and complete follow-up, including list of any outcome data to be collected for participants who discontinue or deviate from intervention protocols	298

Data management	19	Plans for data entry, coding, security, and storage, including any related processes to promote data quality (eg, double data entry; range checks for data values). Reference to where details of data management procedures can be found, if not in the protocol	
Statistical methods	20a	Statistical methods for analysing primary and secondary outcomes. Reference to where other details of the statistical analysis plan can be found, if not in the protocol	285
	20b	Methods for any additional analyses (eg, subgroup and adjusted analyses)	
	20c	Definition of analysis population relating to protocol non-adherence (eg, as randomised analysis), and any statistical methods to handle missing data (eg, multiple imputation)	
**Methods: Monitoring**			
Data monitoring	21a	Composition of data monitoring committee (DMC); summary of its role and reporting structure; statement of whether it is independent from the sponsor and competing interests; and reference to where further details about its charter can be found, if not in the protocol. Alternatively, an explanation of why a DMC is not needed	312
	21b	Description of any interim analyses and stopping guidelines, including who will have access to these interim results and make the final decision to terminate the trial	298
Harms	22	Plans for collecting, assessing, reporting, and managing solicited and spontaneously reported adverse events and other unintended effects of trial interventions or trial conduct	
Auditing	23	Frequency and procedures for auditing trial conduct, if any, and whether the process will be independent from investigators and the sponsor	
**Ethics and dissemination**			
Research ethics approval	24	Plans for seeking research ethics committee/institutional review board (REC/IRB) approval	323

Protocol amendments	25	Plans for communicating important protocol modifications (eg, changes to eligibility criteria, outcomes, analyses) to relevant parties (eg, investigators, REC/IRBs, trial participants, trial registries, journals, regulators)	323
Consent or assent	26a	Who will obtain informed consent or assent from potential trial participants or authorised surrogates, and how (see Item 32)	329
	26b	Additional consent provisions for collection and use of participant data and biological specimens in ancillary studies, if applicable	
Confidentiality	27	How personal information about potential and enrolled participants will be collected, shared, and maintained in order to protect confidentiality before, during, and after the trial	201
Declaration of interests	28	Financial and other competing interests for principal investigators for the overall trial and each study site	340
Access to data	29	Statement of who will have access to the final trial dataset, and disclosure of contractual agreements that limit such access for investigators	
Ancillary and post-trial care	30	Provisions, if any, for ancillary and post-trial care, and for compensation to those who suffer harm from trial participation	
Dissemination policy	31a	Plans for investigators and sponsor to communicate trial results to participants, healthcare professionals, the public, and other relevant groups (eg, via publication, reporting in results databases, or other data sharing arrangements), including any publication restrictions	323
	31b	Authorship eligibility guidelines and any intended use of professional writers	
	31c	Plans, if any, for granting public access to the full protocol, participant-level dataset, and statistical code	
**Appendices**			
Informed consent materials	32	Model consent form and other related documentation given to participants and authorised surrogates	Attached
Biological specimens	33	Plans for collection, laboratory evaluation, and storage of biological specimens for genetic or molecular analysis in the current trial and for future use in ancillary studies, if applicable	

For peer review only

*It is strongly recommended that this checklist be read in conjunction with the SPIRIT 2013 Explanation & Elaboration for important clarification on the items. Amendments to the protocol should be tracked and dated. The SPIRIT checklist is copyrighted by the SPIRIT Group under the Creative Commons "Attribution-NonCommercial-NoDerivs 3.0 Unported" license.

For peer review only
