# Supplementary material for: Evaluation of a novel, multicomponent anxiety management programme for people with intellectual disability: protocol for a mixed-methods, quasi-experimental feasibility study
Source: BMJ Open. 2023 Sep 20;13(9):e078411. doi: 10.1136/bmjopen-2023-078411 (PMC10514669; doi:10.1136/bmjopen-2023-078411)
Supplement: Reviewer comments [file bmjopen-2023-078411.reviewer_comments.pdf]

PEER REVIEW HISTORY

BMJ Open publishes all reviews undertaken for accepted manuscripts. Reviewers are asked to complete a checklist review form (**http://bmjopen.bmj.com/site/about/resources/checklist.pdf**) and are provided with free text boxes to elaborate on their assessment. These free text comments are reproduced below.

ARTICLE DETAILS

**TITLE (PROVISIONAL)**	Evaluation of a novel, multi-component, anxiety management programme for people with intellectual disability: protocol for a mixed-methods, quasi-experimental feasibility study
**AUTHORS**	Acton, Danny; Williams, Jonathan; Woodrow, Ceri; Talbot, Grace; Jones, Steven; Lane, Steven; Jaydeokar, Sujeet

This article was not externally reviewed at BMJ Open. Protocols that have been independently assessed prior to submission to BMJ Open are usually fast-tracked to publication on the grounds that further substantial changes will not be possible. This independent assessment will usually be external, independent review for both a substantial grant award from a non-commercial or government funder and ethics approval.
